# Toward a Better Understanding of Who Is Likely to Be Susceptible to the Effects of Rumination on Obsessive–Compulsive Symptoms: An Explorative Analysis

**DOI:** 10.1007/s41811-024-00228-x

**Published:** 2024-09-03

**Authors:** Karina Wahl, Martin Kollárik, Carlotta V. Heinzel, Stefan Koch, Ulrich Voderholzer, Roselind Lieb

**Affiliations:** 1 Division of Clinical Psychology and Epidemiology, Department of Psychology, University of Basel, Missionsstr. 62a, 4055 Basel, Switzerland; 2 Schoen Clinic Roseneck, Prien Am Chiemsee, Germany; 3 Department of Psychiatry and Psychotherapy, Medical Center, Faculty of Medicine, University of Freiburg, University of Freiburg, Freiburg, Germany

**Keywords:** Unwanted intrusive thoughts, Obsessive–compulsive disorder (OCD), Rumination, Experimental studies

## Abstract

We previously found that rumination maintains obsessive–compulsive (OC) symptoms. Our goal was to explore the moderating roles of three characteristics in the immediate and intermediate effects of rumination on OC symptoms: trait rumination, severity of comorbid depressive symptoms, and the tendency to misinterpret the occurrence of unwanted intrusive thoughts as meaningful. We reanalyzed our previous study’s data and explored in a sample of 145 individuals diagnosed with obsessive–compulsive disorder (OCD) whether any of the three characteristics moderated the observed immediate and intermediate effects of rumination on OC symptoms. Only the tendency to misinterpret unwanted intrusive thoughts moderated the immediate and intermediate effects of rumination on OC symptoms. If this result is confirmed in future studies, individuals with OCD and a high tendency to misinterpret unwanted intrusive thoughts might benefit particularly from supplemental interventions targeting the reduction of excessive rumination.

Excessive rumination about distress has been shown to play a role in the onset and maintenance of several symptoms of mental disorders, including depression, anxiety, bulimia, alcohol abuse, and posttraumatic stress (e.g., Hong, 2007; Kuehner & Weber, 1999; Michl et al., 2013; Moulds et al., 2020; Nolen-Hoeksema et al., 2007; Preston et al., 2021; Rohan et al., 2003; Vanderhasselt et al., 2016). In the context of obsessive–compulsive disorder (OCD), rumination is typically, but not exclusively, activated by the occurrence of obsessive thoughts (OTs), in the sense that the person tries to understand the causes and consequences of these thoughts, for example, “Why me? Why do I have these abhorrent thoughts? What if my partner finds out about it?” (Freeston & Ladouceur, 1997). Cross-sectional studies showed that rumination is very common in OCD and is associated with marked distress (Wahl et al., 2011) and that higher rumination is associated with higher obsessive–compulsive (OC) symptom severity (Heinzel et al., 2021; Raines et al., 2017). Additionally, three experimental studies in our group—two analogue studies (Kollárik et al., 2020; Wahl, et al., 2019) and one study including individuals with OCD (Wahl et al., 2021)—consistently found that rumination had an immediate maintaining effect on the urge to neutralize and the distress associated with unwanted intrusive thoughts^1^ (Kollárik et al., 2020; Wahl, et al., 2019) and an immediate and intermediate maintaining effect on OC symptoms (Wahl et al., 2021).

Our previous experimental study (Wahl et al., 2021) is the basis for the current study, and therefore, it is reported in more detail here. Prior to the allocation of individuals with OCD to one of three experimental groups, an idiosyncratic OT was activated in the laboratory. Participants were subsequently randomly allocated to rumination about OC symptoms, rumination about depressed mood, or distraction. The original research question was whether rumination and distraction have differential effects on OC symptoms (see Wahl et al., 2021). Since this was the first experimental study in individuals with OCD, we decided to examine the combined effects of both types of rumination in comparison to distraction to maximize the chances of finding any effects of rumination compared to distraction.^2^ There was an overall decline of OC symptoms (distress, urge to neutralize, and frequency of OTs) from baseline (prior to rumination/distraction) to the end of the study (after rumination/distraction). However, those individuals who had ruminated (either about OC symptoms or about depressed mood) showed an *attenuated* decline in distress, the urge to neutralize, and frequency of OTs compared to those who were distracted, with medium to large effect sizes. We interpreted this reduced decline as an indicator that rumination has an immediate maintaining effect on OC symptoms.

Additionally, the intermediate effects of rumination were investigated by assessing OC symptoms 2, 4.5, and 24 h after the laboratory experiment using ecological momentary assessment (EMA). The comparison of the combined effects of both types of rumination and distraction did not result in significant differences (see Wahl et al., 2021). However, the study found an interesting differential intermediate effect of focus of rumination: Rumination about OC symptoms resulted in an increase in OC symptom severity compared to rumination about depressed mood 24 h after the end of the experimental session. This was interpreted as rumination about OC symptoms having an intermediate maintaining effect on OC symptoms.

These findings, together with the findings from the two analogue studies (Kollárik et al., 2020; Wahl et al., 2019), raise the question of which characteristics might make an individual prone to these immediate and intermediate effects of rumination on OC symptoms. The identification of relevant characteristics might help practitioners make important clinical decisions about who might profit most from potential supplemental interventions targeting excessive rumination in cognitive behavioral treatment. For example, if we found that individuals with OCD and high levels of comorbid depressive symptoms are particularly prone to the effects of rumination on OC symptoms, then this would indicate that clinicians should pay particular attention to the detrimental effects of rumination in those individuals and use additional interventions targeting excessive rumination accordingly.

In our view, three personal characteristics potentially make individuals with OCD particularly prone to the immediate and intermediate effects of rumination: trait rumination, severity of comorbid depressive symptoms, and the tendency to misinterpret the occurrence of unwanted intrusive thoughts, including obsessions, as meaningful. Trait rumination reflects the tendency to dwell on causes, meanings, and consequences of symptoms (Nolen-Hoeksema et al., 2008). It is plausible to assume that one discrete episode of rumination (e.g., as induced in our laboratory study) might exert its immediate and intermediate influence on OC symptoms particularly in those individuals who generally have a high tendency to ruminate. One could imagine, for example, that a certain threshold must be exceeded before rumination takes effect. This threshold could be reached only if there is a trait tendency to ruminate frequently and extensively. Trait rumination is often used as a moderator in experimental studies (e.g., Quinn et al., 2014).

Higher comorbid depression might exert its moderating influence on one particular episode of rumination through particular depressive symptoms. Along with the well-established reciprocal association between overall depressive symptoms and rumination (e.g., Whisman et al., 2020), there are also specific associations between rumination and particular depressive symptoms such as sleeping problems, lack of energy or concentration, and problems in decision-making (e.g., Pillai et al., 2014). It is conceivable that such depressive symptoms (e.g., being tired, feeling drained of energy) make an individual with OCD particularly prone to the effects of one particular rumination episode.

Finally, the tendency to misinterpret the occurrence of unwanted intrusive thoughts as meaningful reflects dysfunctional beliefs that these thoughts are important in the sense that they might convey some hidden meaning to the self or that experiencing these thoughts might be harmful (Obsessive Compulsive Cognitions Working Group, 2003). It has been suggested that a high tendency to misinterpret unwanted intrusive thoughts as meaningful might make individuals prone to the effects of rumination in the context of OCD (e.g., Raines et al., 2017). The tendency to misinterpret unwanted intrusive thoughts as meaningful can be operationalized in many ways; one common operationalization is thought–action fusion, which refers to thinking that experiencing an unwanted intrusive thought (e.g., about sexually molesting someone) makes it more likely to happen and/or to thinking that experiencing an unwanted intrusive thought is morally equivalent to the corresponding action (Rachman & Shafran, 1999). Conceptually, the tendency to misinterpret unwanted intrusive thoughts as meaningful is usually seen as the more generic term, to which thought–action fusion is subordinate (Jiménez-Ros et al., 2020; Obsessive Compulsive Cognitions Working Group, 2003).

The cognitive model of OCD (e.g., Purdon & Clark, 1993; Rachman, 1998) proposes that the misinterpretation of otherwise harmless but intrusive and unwanted thoughts as signaling danger to the self or others is one of the key factors that determine how distressing OTs develop. Within the cognitive model, thought–action fusion can be seen as a long-standing belief that makes a person vulnerable to catastrophic interpretations or as an ad hoc maladaptive appraisal of the unwanted intrusive thought (Berle & Starcevic, 2005). A large number of studies have shown the relevance of thought–action fusion for OCD (e.g., Hezel et al., 2019; Rassin et al., 2001; Thompson-Hollands et al., 2013). Because of its relevance to OCD, we operationalized the tendency to misinterpret unwanted intrusive thoughts in this study as thought–action fusion.

None of the previous experimental studies aimed to identify potential characteristics that make individuals with OCD prone to the effects of rumination on OC symptoms. To address this gap in the literature, we reanalyzed the data of the study by Wahl et al. (2021) using moderator analysis. Our goal was to explore the moderating roles of three characteristics in the immediate and intermediate effects of rumination on OC symptoms as found in Wahl et al. (2021): trait rumination, severity of comorbid depressive symptoms, and the tendency to misinterpret the occurrence of unwanted intrusive thoughts as meaningful.

## Method

The study is a reanalysis of the study by Wahl et al. (2021). In particular, we focus here exclusively on those previous analyses that showed an effect of rumination on OC symptoms:

- For the immediate effects: the difference between the combined rumination groups in comparison to distraction;
- For the intermediate effects: the difference between rumination about OC symptoms and rumination about depressed mood.

### Participants

Recruitment of potential participants took place at eight inpatient and outpatient clinics specializing in treating OCD in Switzerland and Germany and from a self-help group (Swiss Society for OCD). Inclusion criteria were (a) a primary diagnosis of OCD based on the *Diagnostic and Statistical Manual of Mental Disorders* (text revision, *DSM-IV-TR*; American Psychiatric Association, 2000; German version: Saß et al., 2003), (b) age > 17 years, and (c) the presence of an OT that would likely cause distress when spoken aloud. Exclusion criteria were acute suicidality or self-harm, a current *DSM-IV-TR* substance use disorder, or current *DSM-IV-TR* schizophrenia or other psychotic disorder. One hundred forty-five participants diagnosed with OCD met the inclusion criteria and did not meet the exclusion criteria.

### Measures

#### Assessment of Diagnoses and OC Symptom Severity

Structured Clinical Interview for Mental Disorders (SCID). To assess *DSM-IV-TR* diagnoses on Axis I, including OCD, the German version of the SCID (Wittchen et al., 1997) was administered by trained doctoral students under the supervision of the first author. The SCID has demonstrated acceptable reliability (Lobbestael et al., 2011).

Yale–Brown Obsessive–Compulsive Scale (Y-BOCS). The German version of the Y-BOCS (Hand & Büttner-Westphal, 1991) was used as a clinician rating to assess OC symptom severity. It is a structured clinical interview that is widely recognized as the gold standard for assessing OC symptoms. It consists of two subscales that assess obsessions and compulsions independently with five items each, using information and observations gathered during the interview. Answers are rated on a 5-point Likert scale ranging from 0 to 4, with higher scores indicating higher symptom severity. Additional items provide further information about other aspects of OCD, such as insight into the irrationality of symptoms. The total score is the sum of the 10 items across the two subscales (obsessions and compulsions), ranging from 0 to 40.

Obsessive–Compulsive Inventory, Revised (OCI-R). The German version of the OCI-R (Gönner et al., 2007) was used as a self-report measure to assess OC symptoms. It is an 18-item measure that assesses six OC domains (washing, checking, obsessions, neutralizing, hoarding, and symmetry) over the past month. Responses are rated on a 5-point Likert scale ranging from 0 to 4, with higher scores indicating higher symptom severity. The total score is the sum of all 18 items. The German version of the OCI-R has demonstrated good validity and reliability (Gönner et al., 2007).

#### Assessment of Personal Characteristics (Moderators)

The moderating variables trait rumination, severity of comorbid depressive symptoms, and the tendency to misinterpret the occurrence of unwanted intrusive thoughts as meaningful were assessed with standardized questionnaires before the experimental procedure started.

Response Style Questionnaire (RSQ). The Symptom-Focused Rumination subscale (eight items) of the German version of the RSQ (Bürger & Kühner, 2007) was used to assess trait rumination. Respondents use a 4-point Likert scale (ranging from 1 to 4) to indicate the typical frequency of symptom-related ruminations. The total score is the sum of the eight items, with higher scores indicating higher levels of symptom-related rumination.

Beck Depression Inventory, Revised (BDI-II). The German version of the BDI-II (Hautzinger et al., 2006) was used to assess depressive symptoms. It is a 21-item self-report questionnaire that assesses physical, behavioral, cognitive, motivational, and affective aspects of depression using a 4-point Likert scale (ranging from 0 to 3) over the last 2 weeks. The total score is the sum of all 21 items. The higher the score, the higher the depressive symptoms.

Thought–Action Fusion Scale (TAFS). The tendency to misinterpret unwanted intrusive thoughts as meaningful was operationalized as thought–action fusion (Rachman & Shafran, 1999) and assessed with the German version of the TAFS (Hansmeier et al., 2014). It is a widely used self-report measure with nine items, categorized into TAFS–Moral (experiencing an unwanted intrusive thought is morally equivalent to the corresponding action) and TAFS–Likelihood-self (experiencing an unwanted intrusive thought makes it more likely to happen to oneself) and TAFS–Likelihood-other (experiencing an unwanted intrusive thought makes it more likely to happen to others). Answers are provided on a 5-point scale (0 = *disagree strongly*, 4 = *agree strongly*). The total score is the sum across all items, with higher scores indicating higher thought–action fusion.

#### Assessment of the Outcomes

In the main experimental study, OC symptoms were operationalized as distress associated with the activated OT, the urge to neutralize, and frequency of OTs. Distress (“How distressed are you right now in relation to your obsessive thoughts?”) and urge to neutralize (“To what extent do you feel the urge to neutralize your obsessive thoughts right now?”) were assessed with a 10-point Likert-type scale ranging from 0 (*not at all*) to 9 (*very much so*). The urge to neutralize was defined as “the extent to which participants had the urge to engage in various behaviors to undo the OT, neutralize it, or reduce the associated distress” (Wahl et al., 2021, p. 437). A counter app (Click Counter; FunCoolApps, 2016) on a smartphone was used to assess the immediate effects of rumination on frequency of OTs, with participants being instructed to press the “ + ” button whenever an OT occurred.

During the follow-up period (see the “Procedure” section), OC symptoms were operationalized as self-reported OC symptom severity and were assessed with a modified version of the Y-BOCS using self-developed software.^3^ Participants received three prompts to answer questions on an app, approximately 2 h, 4.5 h, and 24 h after the end of the laboratory experiment. The phrasing of the items assessing the severity of OTs (five items) and compulsions (five items) for the modified Y-BOCS was changed such that (a) only the severity of the activated OT and the associated compulsion were assessed and (b) the referenced time period was the last 30 min. Participants rated each item on a scale of 0 (*minimal severity*) to 4 (*extreme severity*). The sum across all 10 items was used as the OC symptom severity score, separately for each time point.

### Procedure

**Fig. 1:**
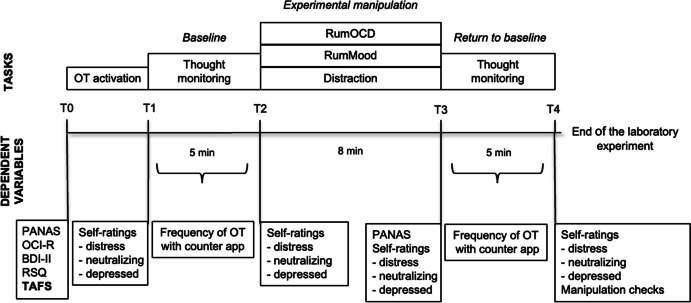
Experimental procedure. BDI-II, Beck Depression Inventory, Revised; counter app, mobile application to count obsessive thoughts (OTs) on a smartphone; OCI-R, Obsessive–Compulsive Inventory, Revised; PANAS, Positive and Negative Affect Scale (Krohne et al., 1996) (The PANAS was administered in the original study but is not reported here.); RSQ, Response Style Questionnaire; RumMood, rumination about mood; RumOCD, rumination about obsessive–compulsive symptoms; T, time point; TAFS, Thought–Action Fusion Scale. The TAFS was not reported in the Wahl et al. (2021) study and is shown in bold. Adapted with permission from Wahl et al. (2021)

Participants were tested individually in the laboratory while seated at a desk with a computer. The experimental procedure is shown in Fig. 1. Having completed informed consent and the set of standardized questionnaires including the assessment of moderating variables (RSQ, BDI-II, TAFS), participants were randomly allocated to one of three experimental groups: rumination about OC symptoms (RumOCD, *n* = 48), rumination about mood (RumMood, *n* = 50), and distraction (*n* = 47). All participants were asked first to write down and then read aloud their most distressing OT (OT activation) and then to record the frequency of their OTs for 5 min with the counter app (baseline). This was followed by 8 min of rumination about OC symptoms, rumination about mood, or distraction (experimental manipulation, see below) and a second 5-min thought-recording phase (return to baseline). Ratings of distress and urge to neutralize were taken at four time points: before the baseline phase (T1), before the experimental manipulation (T2), after the experimental manipulation (T3), and after the return to baseline (T4).^4^

For the follow-up assessments of OC severity, an EMA was used and each participant was provided a smartphone. They were told that they would receive three prompts to answer questions on an app, approximately 2 h (T5), 4.5 h (T6), and 24 h (T7) after the end of the laboratory experiment. Participants returned the smartphones after completing the EMA and were debriefed, and they received pro rata monetary compensation for their time.

#### Experimental Manipulation

The instructions for rumination and distraction were based on a modified version of a widely used paradigm for mood rumination (e.g., Morrow & Nolen-Hoeksema, 1990). Twenty-eight statements were presented to each participant as PowerPoint slides. For the RumOCD group, as reported in Wahl et al., (2021, p. 437),

the statements were modified such that the original references to mood or mental state were replaced with obsessions and compulsions (e.g., ‘*Think about*: what would happen if your current mental state persisted’ was replaced with ‘*Think about*: what would happen if your obsessions and compulsions persisted’) or obsessions and compulsions were added to the original statements (e.g., ‘*Think about*: how passive or active you feel *because of your obsessions and compulsions*’). In this way, the main focus [of rumination] was on [the causes, meanings, and consequences of] OC symptoms. For the RumMood group, the 28 original statements prompting rumination about the causes, consequences, and meaning of the current mood state and typically related depressive symptoms (e.g., ‘*Think about*: how passive or active you feel’) were used (Morrow & Nolen-Hoeksema, 1990). RumMood and RumOCD had an identical number of statements referring to mood/symptoms, consequences, and meanings of the mood/symptoms. The original distraction statements developed by Nolen-Hoeksema and colleagues (Morrow & Nolen-Hoeksema, 1990), focusing on neutral images (e.g., ‘*Think about:* the shadow of a stop sign’), were used for the distraction group.

#### Manipulation Checks

Likert-type scales ranging from 0 (*not at all*) to 100 (*very much so*) were used to check to what degree (a) participants managed to follow the instructions of the statements presented during the experimental manipulation, (b) the statements caused rumination, (c) the focus of the rumination was on OC symptoms, and (d) the focus of the rumination was on mood when the statements were presented.

### Statistical Analysis

#### Manipulation Checks

To analyze differences between the three experimental groups in the degree of focus on instructions and degree to which the statements caused rumination (manipulation checks a and b), one-way analyses of variance (ANOVAs) were used. The homogeneity of variances was tested using Levene’s test. When homogeneity could be assumed, Tukey’s test was applied as a post hoc comparison; otherwise, Games–Howell post hoc tests were used. A 3 × 2 mixed-model ANOVA with group (RumOCD, RumMood, distraction) as a between-subjects factor and focus of rumination (OC symptoms vs. mood) as a within-subject factor was used to analyze the content of rumination (manipulation checks c and d).

#### Moderator Analyses of the Immediate Effects of Rumination

The previous analyses (Wahl et al., 2021) specified a planned contrast that compared the changes from T2 to T3 (the crucial time points immediately before and after the experimental manipulation) in both the RumOCD and the RumMood group (hereafter, combined rumination group) with changes from T2 to T3 in the distraction group, for the outcome variable distress, urge to neutralize, and frequency of OTs. The current analyses extended the previous analyses by exploring whether the immediate effects were moderated by trait rumination (RSQ), comorbid depressive symptoms (BDI-II), or the tendency to misinterpret unwanted intrusive thoughts (TAFS), respectively.

Outliers in the outcome variables (distress, urge to neutralize, and OT frequency) were winsorized, that is, replaced with the next highest values that were not outliers. The interaction effects between the previously specified contrast (changes from T2 to T3 in the combined rumination group vs. distraction) and the potential moderators (RSQ, BDI-II, TAFS) on the outcome variables (distress, urge to neutralize, and OT frequency) were examined, respectively, using the PROCESS model (Hayes et al., 2017). Consistent with Hayes and Cai (2007), we applied the HC4 estimator (Cribari-Neto, 2004) to reduce the potential effects of heteroscedasticity. Predictors were centered prior to the analysis. We report 95% bootstrap confidence intervals to test the interactions on statistical significance and Cohen’s *f*^2^ (Cohen, 1988) as an indicator of effect size for the interactions, where 0.02 is a small effect, 0.15 is a medium effect, and 0.35 is a large effect. A confidence interval that does not include zero is significant. To facilitate interpretation, we depict the results graphically. In particular, we show the effect of a predictor on an outcome at 1 *SD* above and below the mean value of a moderator. For example, we show the effect of the planned contrast on the urge to neutralize at low (1 *SD* below mean), moderate (mean), and high (1 *SD* above mean) TAFS values.

#### Moderator Analyses of the Intermediate Effects of Rumination

In Wahl et al. (2021), multilevel analyses were used to examine the effect of the experimental group (main predictor) on OC symptoms (modified Y-BOCS; outcome) during the follow-up period. A planned contrast was specified that compared the linear changes from T5 to T7 between the RumOCD and RumMood groups. The current analyses extended this analysis by exploring whether the intermediate effects of rumination on OC symptoms are moderated by trait rumination (RSQ), comorbid depressive symptoms (BDI-II), or the tendency to misinterpret unwanted intrusive thoughts (TAFS). In particular, we calculated separate multilevel models to investigate if there was an interaction effect of the specified contrast and moderators on the outcome variables. We visually evaluated the models’ assumptions (functional form, normality of residuals, and homoscedasticity) and detected no violation. Each multilevel model had two levels: measurement occasion (*n* = 3) nested within individuals. We used the maximum likelihood estimation method to estimate the parameters and report unstandardized estimates. The intercept was defined as a random effect for each model; adding a random slope did not improve the goodness-of-fit criteria (Akaike’s and Schwarz’s Bayesian information criteria), so we did not include one. For brevity, we report only the interactions in the “Results” section. To interpret a significant interaction, we plotted the associations between the planned contrast, moderator, and outcome. Following recent studies (Lorah, 2018; Selya et al., 2012), we also calculated Cohen’s *f*^2^ (Cohen, 1988) as an indicator of effect size for the fixed effects.

## Results

### Sample Characteristics

The final number of participants in each group was *n* = 48 for RumOCD, *n* = 46 for RumMood, and *n* = 45 for distraction, since six participants had to be excluded.^5^ Table 1 shows the means and standard deviations of the sample characteristics, test statistics for group differences, and internal consistencies. There were no differences between experimental groups in sociodemographic data (sex, age, marital status, years of education), number of concurrent comorbid disorders, and OC symptoms (Y-BOCS and OCI-R). More participants in the RumOCD group took psychopharmacological medication compared to the other groups. Regarding the moderators, there were no differences in comorbid depressive symptoms (BDI-II) or tendency to misinterpret unwanted intrusive thoughts (TAFS). However, participants in the RumMood group had higher trait rumination (RSQ) than those in the other groups, with a small to medium effect size. Internal consistency (Cronbach’s alpha) of all scales was high to excellent with the exception of the Y-BOCS Obsessions subscale, which showed acceptable consistency.

**Table 1:** Demographics, comorbidity, medication, obsessive–compulsive symptoms (Y-BOCS and OCI-R), moderators, Cronbach’s alpha, test statistics, and effect sizes for differences between experimental groups

Variable	Experimental group			Statistics				
	RumOCD (*n* = 48)	RumMood (*n* = 46)	Distraction (*n* = 45)	*α*	χ^2^ (2)	*p*	*η*_p_^2^	*F* (2136)
Female, *N* (%)	28 (58.33)	30 (65.22)	32 (71.11)		1.67	0.43		
Marital status, *N* (%)					1.03	0.61		
With partner (married or with partner)	21 (43.75)	24 (52.17)	24 (53.33)					
Without partner (single, divorced, widowed)	27 (56.25)	22 (47.83)	21 (46.67)					
Years of education, *N* (%)^a^					7.50	0.06		
9–10	29 (61.70)	19 (43.18)	18 (40.00)					
12–13	18 (38.30)	23 (52.27)	27 (60.00)					
< 9	—	2 (4.55)	—					
Psychopharmacological medication, *N* (%)	41_c_ (85.42)	30_d_ (65.22)	28_d_ (62.22)		7.31	0.03		
Age,* M* (*SD*)	34.44 (11.69)	32.57 (10.99)	35.16 (14.47)			0.59	0.01	0.53
No. concurrent comorbid disorders,* M* (*SD*)	1.52 (1.74)	1.41 (1.38)	1.49 (1.39)			0.94	0.001	0.06
OC symptoms (Y-BOCS: total), *M* (*SD*)	22.33 (6.72)	21.30 (6.11)	19.93 (6.92)	0.84		0.22	0.02	1.55
Obsessions (Y-BOCS: Obsessions scale)	11.29 (3.66)	13.15 (15.54)	10.56 (3.52)	0.72		0.40	0.01	0.93
Compulsions (Y-BOCS: Compulsions scale)	11.02 (3.74)	10.28 (3.72)	9.38 (4.10)	0.81		0.13	0.03	2.12
OC symptoms (OCI-R total), *M* (*SD*)	27.88 (13.32)	28.65 (11.27)	25.00 (12.36)	0.82		0.34	0.02	1.10
Moderators, *M* (*SD*)								
Trait rumination (RSQ)	18.79_c_ (5.25)	21.59_d_ (5.04)	18.80_c_ (4.96)	0.83		0.01	0.06	4.63
Comorbid depressive symptoms (BDI-II)	21.29 (11.60)	25.76 (11.19)	21.47 (10.70)	0.92		0.10	0.03	2.37
Tendency to misinterpret unwanted intrusive thoughts (TAFS)^b^	22.00 (15.94)	28.00 (16.29)	26.84 (17.61)	0.94		0.19	0.02	1.67

Different subscripts (c, d) indicate differences between groups with *p* < 0.05. Adapted with permission from Wahl et al., (2021, Online Supplement)

*BDI-II* Beck Depression Inventory, Revised; *OC* obsessive–compulsive; *OCI-R* Obsessive–Compulsive Inventory, Revised; *RSQ* = Response Styles Questionnaire; *RumMood* rumination about mood; *RumOCD* rumination about OC symptoms; *TAFS* Thought–Action Fusion Scale; *Y-BOCS* Yale–Brown Obsessive–Compulsive Scale

^a^Three missing values

^b^Four missing values

### Manipulation Checks

Table 2 shows means, standard deviations, and statistical results for group comparisons of the manipulation checks. Results show that, on average, participants were able to follow the rumination/distraction instructions to a high degree without any differences between experimental groups. Both rumination instructions resulted in higher degrees of rumination in the rumination groups than found in the distraction group, with a large effect size. The significant interaction between the focus of rumination and the experimental group, with a medium effect size, indicated that the RumOCD group ruminated more on OC symptoms than the RumMood group, and the RumMood group ruminated more on depressive mood than the RumOCD group. In sum, rumination was instigated in both rumination groups to a similar degree and also the focus of the rumination differed as intended.

**Table 2:** Means and standard deviations of manipulation checks and test statistics for differences between experimental groups

Variable	Experimental group			*p*	*η*_p_^2^	*F* (2136)
	RumOCD (*n* = 48)	RumMood (*n* = 46)	Distraction (*n* = 45)			
Ability to follow instructions of statements	73.75 (20.64)	72.37 (24.64)	80.87 (16.12)	0.12	0.03	2.19
Degree of rumination caused by statements	73.10_c_ (18.93)	72.15_c_ (24.45)	49.40_d_ (31.68)	< 0.001	0.16	12.73
Focus of rumination^a^				0.003^a^	0.08^a^	5.94^a^
OC symptoms	68.06 (27.72)	57.50 (30.13)	37.27 (28.77)			
Depressive mood	47.50 (35.30)	53.59 (31.66)	35.67 (29.31)			

Different subscripts (c, d) indicate differences between groups with *p* < 0.05. Adapted with permission from Wahl et al., (2021, Online Supplement)

*OC* obsessive–compulsive, *RumMood* rumination about mood, *RumOCD* rumination about OC symptoms

^a^Interaction between the focus of rumination and the experimental group

### Moderator Analyses of the Immediate Effects of Rumination

**Fig. 2:**
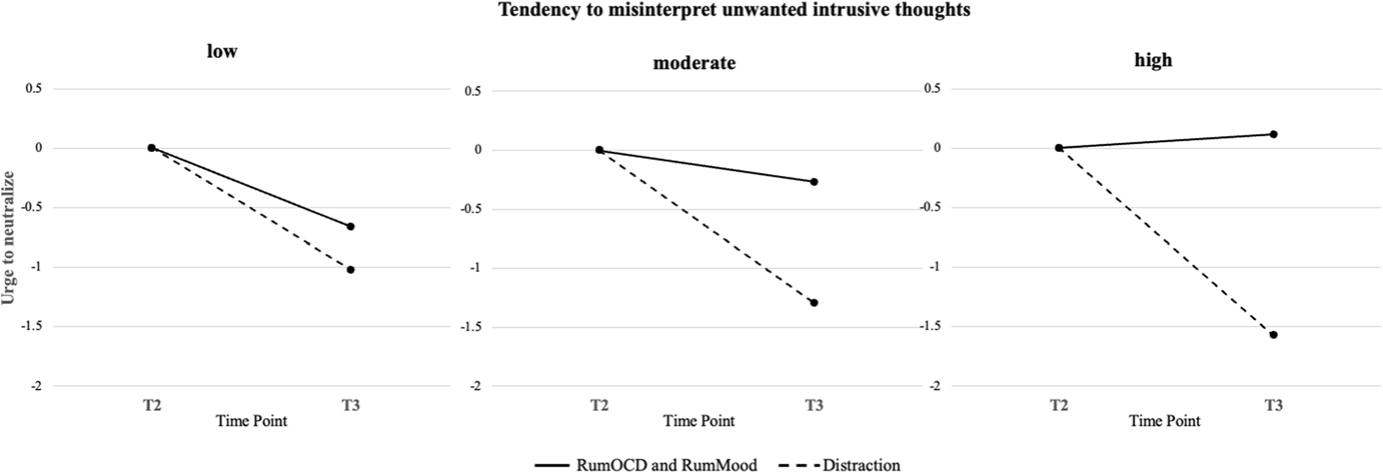
Graphical depiction of the immediate moderator analyses. T2 = Immediately before the experimental manipulation; T3 = immediately after the experimental manipulation; low tendency to misinterpret unwanted intrusive thoughts = 1 *SD* below mean value of the Thought–Action Fusion Scale (TAFS); moderate tendency to misinterpret unwanted intrusive thoughts = mean value of the TAFS; high tendency to misinterpret unwanted intrusive thoughts = 1 *SD* above mean value of the TAFS; RumMood = rumination about mood; RumOCD = rumination about obsessive–compulsive symptoms

Table 3 shows the results of the moderator analysis. Tendency to misinterpret unwanted intrusive thoughts (TAFS) moderated the immediate effect of the planned contrast on urge to neutralize. The effect size for this moderation was small to medium. Figure 2 shows the interaction graphically when the moderator variable tendency to misinterpret unwanted intrusive thoughts (TAFS) is divided into three arbitrary categories, in this case into 1 *SD* below the mean value of the TAFS, the mean value of the TAFS, and 1 *SD* above the mean value of the TAFS. It suggests that as the tendency to misinterpret unwanted intrusive thoughts increased, so too did the differential effect of the combined rumination group compared to the distraction group. Tendency to misinterpret unwanted intrusive thoughts did not moderate the effect of the planned contrast on distress or OT frequency. Trait rumination (RSQ: symptoms) and comorbid depressive symptom severity (BDI-II) did not moderate the effects of the planned contrast on distress, urge to neutralize, or OT frequency.

**Table 3:** Moderator analysis of the immediate effects on outcomes: interactions between the planned contrast and moderators

Interaction of the planned contrast with the moderators	Distress			Urge to neutralize			Obsessive thought frequency		
	*b* (*SE*)	95% bootstrap CI	*f*^2^	*b* (*SE*)	95% bootstrap CI	*f*^2^	*b* (*SE*)	95% bootstrap CI	*f*^2^
Trait rumination (RSQ)	0.08 (0.06)	[− 0.02, 0.21]	0.01	0.04 (0.07)	[− 0.08, 0.18]	0.00	0.18 (0.43)	[− 0.58, 1.03]	0.00
Comorbid depressive symptoms (BDI-II)	0.03 (0.03)	[− 0.02, 0.08]	0.01	0.002 (0.03)	[− 0.05, 0.06]	0.00	0.06 (0.25)	[− 0.33, 0.49]	0.00
Tendency to misinterpret unwanted intrusive thoughts (TAFS)	0.02 (0.02)	[− 0.01, 0.06]	0.01	0.04 (0.02)	[0.001, 0.09]	0.03	0.09 (0.12)	[− 0.13, 0.34]	0.01

Planned contrast: Comparison between the combined rumination group (RumOCD and RumMood) and the distraction group. Moderators: Depressive symptoms, OC symptom severity, trait rumination, thought–action fusion. Outcomes: Distress, urge to neutralize, and obsessive thought frequency

*BDI-II* Beck Depression Inventory, Revised; *CI* confidence interval; *OC* obsessive–compulsive; *RSQ* Response Style Questionnaire; *RumMood* rumination about mood; *RumOCD* rumination about OC symptoms; *TAFS* Thought–Action Fusion Scale

### Moderator Analyses of the Intermediate Effects of Rumination

**Fig. 3:**
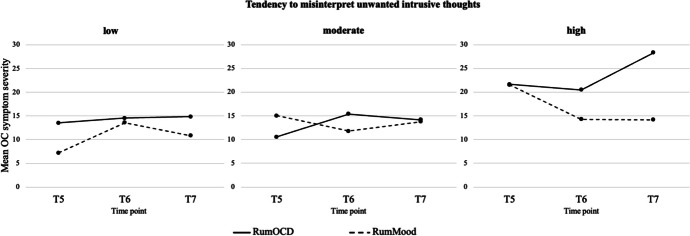
Graphical depiction of the interaction between the planned contrast, time, and tendency to misinterpret unwanted intrusive thoughts, for the intermediate effects of rumination on OC symptoms. Planned contrast: Comparison between the RumOCD and the RumMood groups. OC, obsessive–compulsive; RumMood, rumination about mood; RumOCD, rumination about OC symptoms; low tendency to misinterpret unwanted intrusive thoughts = 1 *SD* below mean value of the Thought–Action Fusion Scale (TAFS); moderate tendency to misinterpret unwanted intrusive thoughts = mean value of the TAFS; high tendency to misinterpret unwanted intrusive thoughts = 1 *SD* above mean value of the TAFS

Table 4 shows the results of the multilevel analysis for the intermediate moderating effects. There was a positive significant interaction effect between the planned contrast (rumination about OC symptoms vs. rumination about mood), time, and tendency to misinterpret unwanted intrusive thoughts (TAFS) on OC symptoms (modified Y-BOCS), with a small to medium effect size. Figure 3 suggests that the interaction between type of rumination and time point was particularly pronounced for those with high levels of tendency to misinterpret unwanted intrusive thoughts. Only participants with high levels experienced an increase in OC symptoms over time in the RumOCD group compared to the RumMood group. There was no interaction effect between the planned contrast, time, and the other moderators (trait rumination, comorbid depressive symptoms) on OC symptoms.

**Table 4:** Moderator analysis of the intermediate effects on OC symptoms: interactions between the planned contrast, time, and moderators

EMA predictor (fixed effect)	OC symptoms (modified Y-BOCS)	*p*
	Coefficient (*SE*)	
Contrast * Time * Trait Rumination (RSQ)	− 0.08 (0.20)	0.710
Contrast * Time * Comorbid Depressive Symptoms (BDI-II)	0.02 (0.10)	0.814
Contrast * Time * Tendency to Misinterpret Unwanted Intrusive Thoughts (TAFS)	0.13 (0.07)	0.048

Planned contrast: Comparison between RumOCD and RumMood groups. Moderators: Trait rumination, comorbid depressive symptoms, tendency to misinterpret unwanted intrusive thoughts. RumOCD is the reference group for the interaction. For example, for the interaction effect of Contrast * Time * Tendency to Misinterpret Unwanted Intrusive Thoughts on OC symptoms, the value 0.13 is the value of rumination about OC symptoms compared to rumination about mood

*BDI-II* Beck Depression Inventory, Revised; *EMA* ecological momentary assessment; *OC* obsessive–compulsive; *RSQ* Response Style Questionnaire; *RumMood* rumination about mood; *RumOCD* rumination about OC symptoms; *TAFS* Thought–Action Fusion Scale; *Y-BOCS* Yale–Brown Obsessive–Compulsive Scale.

## Discussion

The study investigated the role of three personal characteristics as moderators of the effects of rumination on OC symptoms using explorative moderator analyses: trait rumination, severity of comorbid depressive symptoms, and the tendency to misinterpret the occurrence of unwanted intrusive thoughts as meaningful. The tendency to misinterpret unwanted intrusive thoughts as meaningful emerged as a moderator. The higher the tendency to misinterpret, the more pronounced was the difference between rumination and distraction. In particular, if the tendency to misinterpret unwanted intrusive thoughts was low, there was little difference between rumination and distraction in maintaining the urge to neutralize. However, if the tendency to misinterpret was moderate or high, then rumination had a greater effect than distraction. Why the moderating effect was apparent only for urge to neutralize, with a small to medium effect size, and not for distress or frequency of OTs, with very small and nonsignificant effects, remains a curious finding at this stage. Clinical observations by the first author are consistent with the finding that neutralizing is the response that is most susceptible to misinterpretation, more so than distress or OT frequency. This assumption is consistent with cognitive conceptualizations of OCD (e.g., Rachman, 1998).

For the moderation of the intermediate effects of rumination on OC symptoms, we found a similar pattern to that of the immediate effects. The tendency to misinterpret unwanted intrusive thoughts moderated the effects on OC symptoms in the same way as in the immediate term: That is, the differential effects between rumination on OC symptoms and rumination on mood increased with increasing levels of the tendency to misinterpret unwanted intrusive thoughts. Put differently, only if individuals tended to misinterpret unwanted intrusive thoughts as meaningful to a high degree did rumination about OC symptoms maintain OC symptoms more than rumination about mood. Thus, it seems that individuals with moderate to high levels of the tendency to misinterpret unwanted intrusive thoughts as meaningful are particularly vulnerable to the effects of rumination on OC symptoms.

In neither the immediate nor the intermediate moderation analyses did trait rumination or comorbid depressive symptom severity moderate the effects of rumination on OC symptoms. This means that rumination exerts an influence on OC symptoms irrespective of levels of trait rumination and comorbid depressive symptoms. It appears then that individuals with OCD and additionally high levels of trait rumination or comorbid depressive symptoms are not particularly prone to the effects of rumination. Especially, the lack of a moderating effect of level of trait rumination appears counterintuitive at first, given that trait rumination is associated with OC symptom severity (e.g., Heinzel et al., 2021) and also plays a role in the development and maintenance of a variety of mental disorders (e.g., Nolen-Hoeksema, 2000). At second glance, it seems plausible that distinct episodes of rumination (as in our study) and trait rumination (as in Heinzel et al., 2021) might exert their influences on OC symptoms independently. In particular, getting stuck in one discrete ruminative episode might maintain OC symptoms directly. But habitual rumination—maybe as an underlying cognitive tendency that is longer lasting but possibly not as intense as one particular episode—is also associated with OC symptoms, although the particular mechanisms are still unclear.

Why might particularly the tendency to misinterpret unwanted intrusive thoughts as meaningful make the individual vulnerable to the effects of rumination on OC symptoms? One might speculate that the global tendency to misinterpret unwanted intrusive thoughts as meaningful operates as a kind of amplifier for the effects of rumination. Typical ruminative thoughts (“What do these abhorrent thoughts mean? What do they reveal about me as a person? Why, of all people, is it me who has these horrible thoughts?”) share similarities particularly with those misinterpretations of unwanted intrusive thoughts that see the occurrence of the thoughts as an indication of the person’s dangerousness or moral misconduct. Against this background, the effects of rumination about OTs could be more intense and more persistent, with a higher emotional impact. Several previous studies have shown that thought–action fusion is likely to be involved in OCD (e.g., Hezel et al., 2019; Rassin et al., 2001; Thompson-Hollands et al., 2013). Our study supports these previous findings and our results indicate that thought–action fusion not only might directly influence the development and/or maintenance of OC symptoms but might also indirectly exacerbate them by interacting with discrete episodes of rumination.

The present findings should be interpreted in light of the analyses’ limitations. Importantly, all analyses were explorative, since the original study was not powered to conduct moderator analyses. This could mean that small effects might not have been detected in our analyses owing to a lack of power. It is also possible that our findings are spurious and therefore it is key that future studies address the question of which factor make individuals vulnerable to the effects of rumination directly. The psychometric qualities of the EMA measure (modified Y-BOCS) are unknown. Although we used a well-established and effective paradigm to induce rumination in the laboratory, the ecological validity of our findings might be compromised by the standardized, laboratory setting. It remains unclear to what extent our findings can be transferred to naturally occurring everyday-life rumination.

To conclude, our explorative analysis identified the tendency to misinterpret unwanted intrusive thoughts, but neither of the other investigated characteristics (trait rumination, comorbid depressive symptoms), as a factor that might make individuals with OCD particularly prone to the effects of rumination on OC symptoms. If confirmed in future studies, this might indicate that in particular, those individuals with OCD and a high tendency to misinterpret unwanted intrusive thoughts might benefit from supplemental interventions targeting the reduction of excessive rumination in cognitive behavioral therapy. Such interventions could, for example, include adopting a more accepting attitude toward one’s obsessive symptoms (e.g., Külz et al., 2019) or computerized interpretation modification training adapted to rumination in OCD (Hirsch et al., 2020).

## Data Availability

The data sets generated and/or analyzed during the current study are not publicly available owing to lack of permission by the ethics committee but are available from the corresponding author on reasonable request.

## Footnotes

1 “Unwanted intrusive thoughts” is typically used as an umbrella term for unpleasant thoughts that intrude into one’s consciousness, ranging from common thoughts that almost everybody experiences occasionally to repugnant OTs characteristic of OCD (e.g., Clark & Radomsky, 2014). In the context of OCD-related experimental studies, unwanted intrusive thoughts are often operationalized as thoughts about a potential future accident, for example, about a beloved person being involved in a car accident (e.g., van den Hout et al., 2002). They are considered analogues to OTs in the sense that they are also perceived as negative and intrusive and result in an urge to neutralize, although to a lesser extent and without the marked impairment that is typical of obsessions.

2 Additionally, in a second step, the differential effects of rumination about OC symptoms versus rumination about depressed mood were investigated; for results, see Wahl et al. (2021).

3 Additionally, positive and negative affect were assessed, but results are not reported here since we focus on OC symptoms as outcomes.

4 Additionally, depressed mood was assessed with visual analogue scale ratings, but results are not reported here since we focus on OC symptoms as outcomes.

5 Two participants did not experience any distress during thought activation; two other participants’ activated thoughts were not typical of OCD but were consistent with worrisome thoughts; technical problems occurred with one participant; and for one other, more than 63% of the data was missing.
